# A renally clearable tumor-targeted probe enabling functional delineation of tumor margins by NIR-II fluorescence lifetime imaging

**DOI:** 10.1016/j.mtbio.2026.103274

**Published:** 2026-05-23

**Authors:** Jin Zhang, Jiuling Liao, Dong Han, Xingsheng Ren, Dehong Hu, Duyang Gao, Pengfei Zhang, Shengnan Yuan, Wei Zheng, Christopher J. Butch, Bo Dai, Huiming Cai, Yiqing Wang, Hairong Zheng, Zonghai Sheng

**Affiliations:** a Research Center for Advanced Detection Materials and Medical Imaging Devices, Institute of Biomedical and Health Engineering, Shenzhen Institute of Advanced Technology, Chinese Academy of Sciences, Shenzhen, 518055, PR China; b State Key Laboratory of Biomedical Lmaging Science and System, Shenzhen, 518055, PR China; c Research Center for Biomedical Optics and Molecular Imaging, Shenzhen Key Laboratory for Molecular Imaging, Guangdong Provincial Key Laboratory of Biomedical Optical Imaging Technology, Shenzhen Institutes of Advanced Technology, Chinese Academy of Sciences, Shenzhen, 518055, PR China; d Department of Biomedical Engineering, College of Engineering and Applied Sciences, State Key Laboratory of Analytical Chemistry for Life Science, Nanjing University, Nanjing, PR China; e Guangdong Key Laboratory of Nanomedicine, Chinese Academy of Sciences-Hong Kong Joint Lab for Biomaterials, Chinese Academy of Sciences Key Laboratory of Biomedical Imaging Science and System, Institute of Biomedicine and Biotechnology, Shenzhen Institutes of Advanced Technology, Chinese Academy of Sciences, Shenzhen, 518055, PR China; f Nanjing Nuoyuan Medical Devices Co., Ltd, Nanjing, PR China; g Department of Thoracic Surgery, Nanjing Drum Tower Hospital, School of Medicine, Nanjing University, Nanjing, PR China

**Keywords:** NIR-II fluorescence lifetime imaging, Molecular probe, Tumor margins delineation, Renally clearable

## Abstract

Accurate intraoperative delineation of tumor margins remains a major clinical challenge because conventional fluorescence imaging is strongly influenced by probe concentration, tissue heterogeneity, and imaging conditions. Here, we report a renally clearable, tumor-targeted molecular probe, NY-07, for quantitative tumor-margin delineation using second near-infrared (NIR-II) fluorescence lifetime (FLT) imaging. NY-07 is constructed by conjugating a cyanine-based fluorophore with pemetrexed as an active targeting ligand, yielding a clinically compatible probe with excellent solubility, high optical stability, and efficient renal clearance. NY-07 exhibits tail emission in the NIR-II window with a stable fluorescence lifetime of 480 ± 0.6 ps and NIR-II brightness approximately twice that of indocyanine green (ICG) at the same concentration, while retaining ∼70% of its NIR-II signal after 7 days of storage. In vivo imaging demonstrates rapid renal excretion, with strong kidney and bladder signals within seconds after injection and minimal hepatic accumulation. Using cell models, tumor-bearing mice, and patient-derived tissues, we show that NY-07 enables target-specific NIR-II FLT imaging and achieves quantitative discrimination between tumor, inflammation, and normal tissues based on distinct lifetime signatures, enabling precise visualization of tumor margins independent of probe concentration and imaging parameters. Compared with conventional NIR-II fluorescence intensity (FLI) imaging, FLT imaging significantly reduces false-positive signals and improves boundary definition in complex biological environments. This work establishes a clinically translatable strategy that integrates renal clearance, active tumor targeting, and lifetime-resolved imaging for functional tumor-margin delineation, providing a robust platform for improving surgical precision and advancing next-generation image-guided oncologic surgery.

## Graphical abstract

Schematic diagram illustrating the advantages of NIR-II fluorescence lifetime imaging with targeted probe NY-07 for quantitative tumor margins delineation compared to traditional fluorescence intensity imaging.

**Figure:**
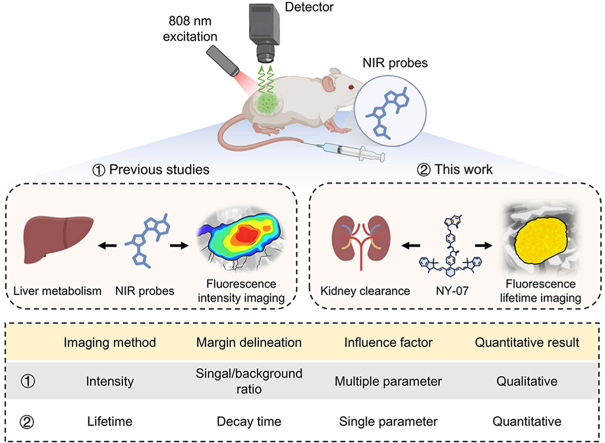

## 1 Introduction

Accurate intraoperative delineation of tumor margins is critical for improving surgical resection efficiency, protecting normal tissues and functions, and minimizing postoperative recurrence [1,2]. Compared to the first near-infrared (NIR-I, 700–900 nm) fluorescence imaging, the NIR-II (1000–1700 nm) provides enhanced tissue penetration depth, reduced autofluorescence from biological tissues, and a higher signal-to-background ratio (SBR) [3,4]. These advantages enable more precise visualization of tumor margins within the so-called “optical transparency window.” To date, various NIR-II fluorescent probes has been developed for delineation of tumor margins, including quantum dots [5,6], rare-earth–doped nanocrystals [[7], [8], [9]], gold clusters [10,11], aggregation-induced emission dots [[12], [13], [14]], conjugated polymers [15,16], and small-molecule dyes [17,18]. However, the accuracy of intraoperative margin identification remains influenced by multiple factors such as probe brightness, intratumoral distribution, and imaging parameters [19,20].

In contrast to conventional FLI imaging, FLT imaging enables quantitative dection of probe–target interactions and is inherently independent of probe concentration and imaging parameters [21,22]. These characteristics offer opportunities to reduce false-positive signals and to achieve quantitative delineation of tumor margins [23,24]. Recently, Zhang and colleagues reported multiplexed NIR-II FLT imaging based on rare-earth luminescent nanocrystals [25], highlighting the promise of this imaging modality for tumor margin visualization. Our group further demonstrated that a tumor-targeted albumin–indocyanine green (ICG) probe enables more accurate tumor margin identification using FLT imaging than FLI imaging [26]. Despite recent progress, complex metabolism, safety concerns, and regulatory of probes limit the clinical translation of FLT imaging.

**Fig. 1:**
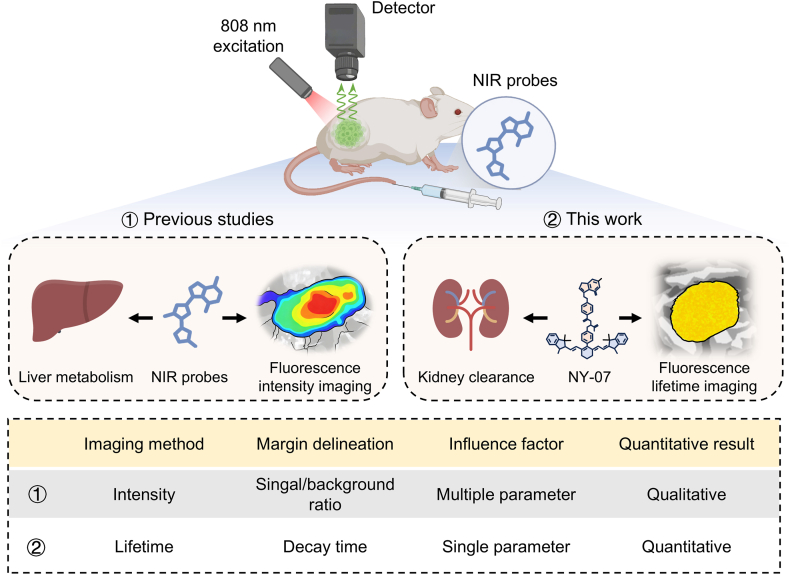
**Schematic of the NIR-II FLT targeted molecule probe NY 07 for quantitative visualization of tumor margins.** Conventional liver-metabolized fluorescence probes rely on intensity-based imaging for tumor margin delineation, which is susceptible to multiple interfering factors and exhibits an intensity-dependent characteristic. In contrast, the NIR-II FLT targeted small molecule probe NY 07 developed in this study is primarily eliminated via renal clearance. When combined with NIR-II FLT imaging technology, the NY 07 probe enables more accurate and quantitative visualization of tumor margins.

Here, we report a renal-clearable FLT imaging probe, NY-07, for quantitative tumor-margin delineation (Fig. 1). NY-07 was constructed by conjugating an ICG derivative with pemetrexed as an active targeting ligand. Notably, NY-07 has obtained clinical trial authorization from both NMPA and FDA for applications utilizing NIR-I FLI imaging. We demonstrate that NY-07, with its tail-fluorescence emission in NIR-II window, exhibits distinct and stable FLT imaging properties. Its optical stability, solubility, and imaging depth were systematically evaluated both in vitro and in vivo. Using cell cultures and tumor-bearing mice, we show that NY-07 enables target-specific NIR-II FLT imaging and quantitatively delineates tumor margins through differential lifetime signatures between tumor, inflammation and normal tissues.

## 2 Results and discussion

### 2.1 NIR-II FLI and FLT properties of NY-07 probe

**Fig. 2:**
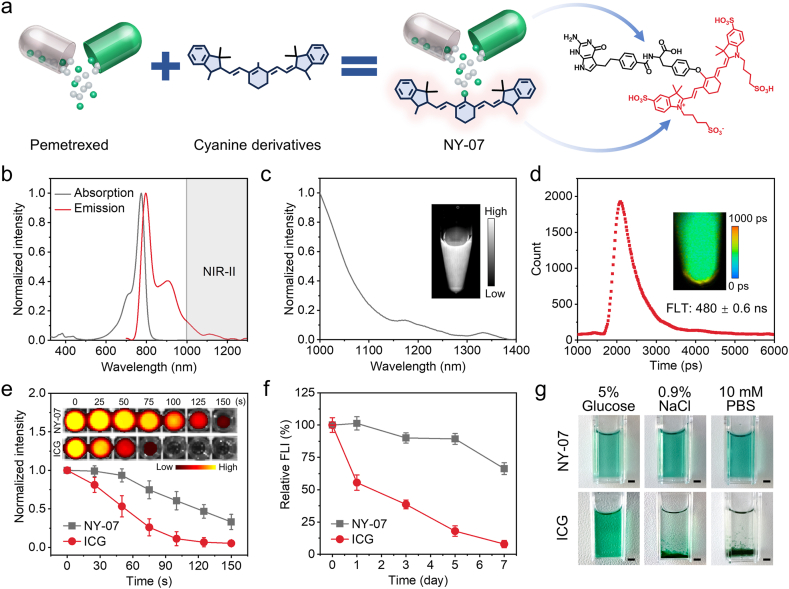
**Characterization of the NY-07 molecular probe.** (a) Schematic illustration of NY-07 synthesis. Pemetrexed serves as the targeting ligand, and a cyanine derivative acts as the fluorophore; the two components are covalently linked via Williamson ether synthesis. (b) The UV–vis–NIR absorption and fluorescence emission spectra of NY-07 (λex = 660 nm). The grey shaded region denotes the NIR-II window. (c) NIR-II fluorescence emission spectrum of NY-07. Inset: NIR-II fluorescence image of a 10 μg mL^−1^ NY-07 solution excited at 808 nm and collected with a 1075 nm long-pass filter. (d) NIR-II fluorescence decay profile of a 10 μg mL^−1^ NY-07 solution. Inset: corresponding two-dimensional FLT image (λex = 808 nm; λem > 1000 nm). (e) Photobleaching behavior of NY-07 and ICG under continuous 808 nm laser irradiation (10 μg mL^−1^). Inset, fluorescence images acquired after different irradiation times (laser power density, 0.4 W cm^−2^; λex = 745 nm; λem = 820 nm). (f) NIR-II FLI imaging variation of NY-07 and ICG during storage (2 μg mL^−1^). (g) Photographs of NY-07 and ICG solutions (200 μg mL^−1^) after 2 days of storage in different solvents.

NY-07 was synthesized by covalently conjugating pemetrexed as the targeting ligand with an ICG derivative as the fluorophore (Fig. 2a and S1) according to previous literature [27]. The molecular structure and molecular weight of NY-07 were verified using mass spectrometry (Fig. S2) and nuclear magnetic resonance spectroscopy (Fig. S3), respectively. UV–vis and fluorescence spectral analyses revealed an absorption maximum at 774 nm and an emission peak at 795 nm (Fig. 2b). Notably, NY-07 demonstrated a significant long-wavelength emission tail that extended into the NIR-II window (Fig. 2c). Furthermore, NIR-II fluorescence brightness of NY-07 was higher than free ICG (a small molecule probe for clinical use) at the same concentration (Fig. S4), indicating its great potential for NIR-II FLI imaging. Time-resolved measurements revealed that the NIR-II fluorescence lifetime of NY-07 was 480 ± 0.6 ps (Fig. 2d), which is comparable to that of ICG (Fig. S5) and substantially shorter than that reported for rare-earth-doped nanocrystals [25], facilitating rapid FLT imaging with reduced acquisition times. NY-07 also demonstrated significantly improved photostability (Fig. 2e). Under continuous 808 nm laser irradiation, NY-07 showed markedly reduced photobleaching compared with free ICG. After a 7 days storage period at ambient temperature under light-protected conditions, NY-07 maintained approximately 70% of its original fluorescence intensity, whereas free ICG retained only about 10%, indicating superior storage stability of NY-07. The solubility of NY-07 was further evaluated in commonly used buffer systems and clinically relevant injection solutions, including 5% glucose solution, 0.9% saline, and 10 mM phosphate-buffered saline (PBS). NY-07 exhibited good solubility in all tested media, with no detectable aggregation or precipitation, whereas free ICG showed visible precipitation in normal saline and PBS (Fig. 2g).

### 2.2 Imaging depth of the NY-07 probe in the NIR-I and NIR-II windows

**Fig. 3:**
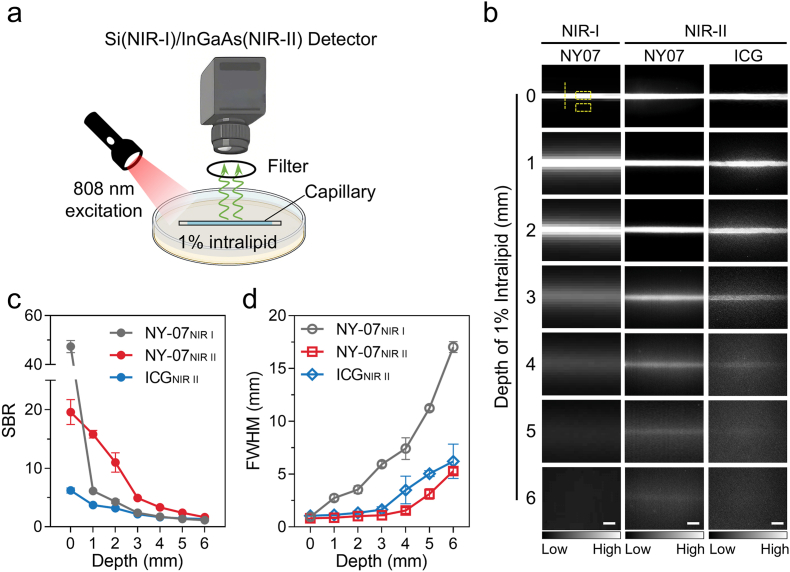
**Imaging depth of NY-07 in Intralipid phantoms.** (a) Schematic illustration of NIR-I and NIR-II FLI imaging of the NY-07 probe confined in a capillary tube and embedded at specific depths within a 1% Intralipid phantom. (b) Representative NIR-I and NIR-II FLI images of NY-07 and ICG in 1% Intralipid at progressively increasing depths. Both NY-07 and ICG were used at concentrations of 7.63 μM. The imaging parameters were as follows: for NIR-I, excitation wavelength (λex) was 745 nm and emission wavelength (λem) was 820 nm; for NIR-II, excitation wavelength was 808 nm, with signal collection through a 1000 nm long-pass filter. Exposure time was 1 s. Fluorescence intensity was quantified using ImageJ. The SBR was calculated from regions of interest indicated by yellow boxes, and the full width at half maximum (FWHM) was derived from intensity profiles along the yellow dashed lines. (c,d) Quantitative analysis of SBR and FWHM as a function of phantom thickness. n = 3. (For interpretation of the references to colour in this figure legend, the reader is referred to the Web version of this article.)

Tissue-mimicking phantoms composed of 1% fat emulsion was used to evaluate the imaging depth of NY-07 in the NIR-I and NIR-II windows (Fig. 3a). In both models, the fluorescence signal of NY-07 gradually attenuated with increasing tissue depth (Fig. 3b). Notably, NY-07 achieved a maximum imaging depth of 6 mm in the NIR-II region, exceeding the 4 mm depth obtained in the NIR-I region. Quantitative assessments demonstrated that NY-07 consistently displayed a greater SBR within the NIR-II spectral region compared to the NIR-I region at all tested depths (Fig. 3c). As the depth increased from 1 mm to 6 mm, the full width at half maximum (FWHM) in the NIR-II window stayed under 2 mm, while in the NIR-I window it expanded to 5 mm. Furthermore, both the imaging depth and SBR achieved by NY-07 in the NIR-II window surpassed those of the clinically utilized indocyanine green (ICG) under identical conditions. This observation confirms the superior potential of NY-07 for NIR-II imaging applications.

### 2.3 In vitro active targeting imaging of NY-07

**Fig. 4:**
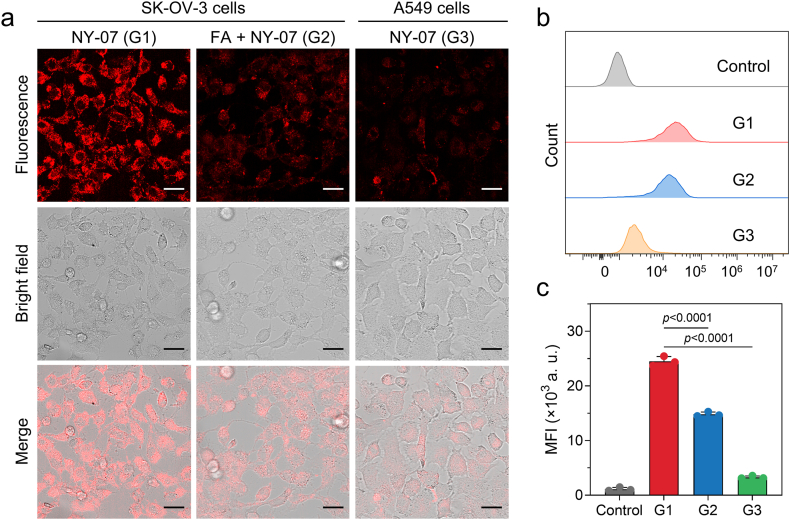
**In vitro tumor-targeting performance of NY-07.** (a) Confocal fluorescence images of FRα-overexpressing SK-OV-3 cells incubated with NY-07 in the absence or presence of excess folic acid (FA) for competitive blockade. FRα-low A549 cells were used as a negative control. The NY-07 concentration was 30 nM, and the incubation time was 2 h. Scale bar: 30 μm. (b) Flow cytometry analysis of cellular fluorescence intensity in SK-OV-3 cells with or without FA blockade and in A549 cells after incubation with NY-07 (30 nM, 2 h). (c) Quantification of flow cytometry data. Data are presented as mean ± standard deviation (s.d.). Statistical significance was assessed using a two-tailed Student's t-test.

To assess the active targeting ability of NY-07, SK-OV-3 ovarian cancer cells, which exhibit high FRα expression, and A549 cells, characterized by low FRα expression, were employed as in vitro models. Three groups were examined: G1 (SK-OV-3 + NY-07), G2 (SK-OV-3 cells pretreated with folic acid (FA) + NY-07), and G3 (A549 + NY-07). After 3 h of incubation, confocal fluorescence imaging revealed strong cytoplasmic fluorescence in SK-OV-3 cells in G1 (Fig. 4a). In contrast, fluorescence intensity was significantly diminished in G2 due to competitive inhibition by FA, whereas only faint signals were detected in the FRα-low A549 cells (G3). These results indicate that cellular uptake of NY-07 is predominantly mediated by FRα. Furthermore, flow-cytometry analysis demonstrated that the mean fluorescence intensity (MFI) in SK-OV-3 cells of G1 was approximately 1.7-fold higher than in G2 cells and 7.4-fold higher than that in G3 cells (Fig. 4b and c), confirming the high specificity of NY-07 toward FRα-mediated active targeting.

Furthermore, in vitro FLT imaging was performed on FRα-overexpressing SK-OV-3 cells and FRα-low A549 cells following incubation with NY-07. The results (Fig. S6) showed that the fluorescence lifetime in SK-OV-3 cells (0.75 ± 0.04 ns) was significantly longer than that in A549 cells (0.48 ± 0.03 ns). These findings directly demonstrate that specific binding of NY-07 to FRα is a key factor responsible for the observed prolongation of its fluorescence lifetime. Meanwhile, the cytotoxicity of NY-07 also was evaluated using the CCK-8 assay. SK-OV-3 cells and A549 cells were incubated with various concentrations of NY-07 (0-200 μM) for 24 h. The results (Fig. S7) showed that even at the highest concentration (200 μM), the cell viability of both cell lines remained above 90%, indicating that NY-07 exhibits good in vitro biocompatibility.

### 2.4 Renal clearance of NY-07

**Fig. 5:**
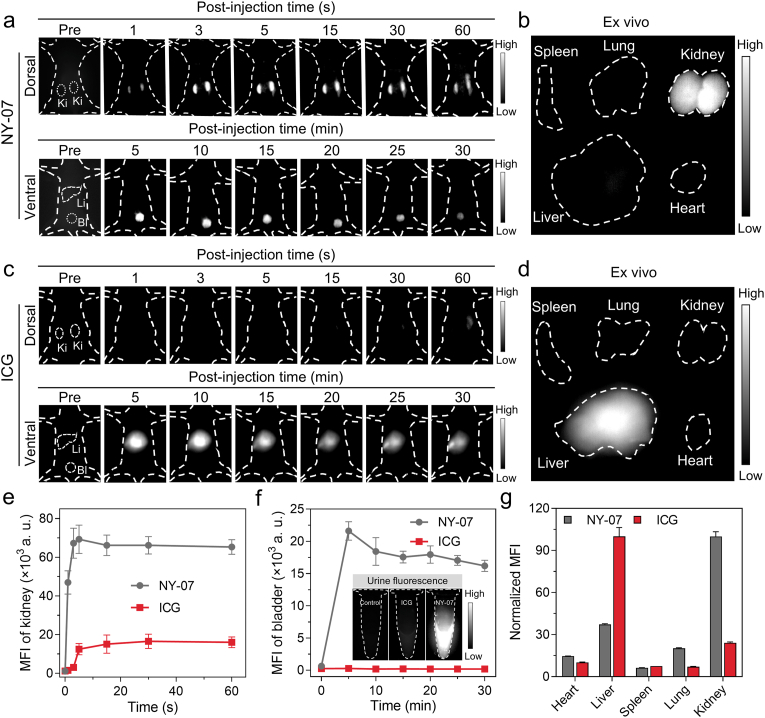
**In vivo renal clearance and biodistribution of NY-07.** (a) Representative whole-body NIR-II FLI images of healthy mice captured in dorsal and ventral positions at specified time points following intravenous injection of NY-07. (b) Representative ex vivo NIR-II FLI images of major organs (heart, liver, spleen, lungs, and kidneys) and tumor harvested 48 h post-injection. (c) Representative whole-body NIR-II FLI images of mice in dorsal and ventral positions at specified time points following intravenous injection of ICG. (d) Corresponding ex vivo NIR-II FLI images of major organs and tumor collected 48 h post-injection. NY-07 and ICG were administered via tail vein injection at an equivalent dose of 10 nmol each. (e) Time-dependent changes in mean MFI within the left and right kidney regions following administration of NY-07 or ICG. (f) Time-dependent changes in MFI within the bladder region following administration of NY-07 or ICG. Inset: Representative NIR-II FLI images of urine samples collected from treated mice. (g) Quantification of MFI in excised organs and tumor at 48 h post-administration of NY-07 or ICG. NIR-II FLI imaging was quantified using ImageJ. For all imaging experiments, excitation was performed at 808 nm and emission was collected using a 1000 nm long-pass filter. Data are presented as mean ± s.d. (n = 3).

The dynamic distribution of NY-07 in Balb/C mice following intravenous administration was monitored in real time using NIR-II fluorescence imaging. As illustrated in Fig. 5a, a distinct fluorescence signal emerged rapidly in the renal region of the mouse dorsal view shortly after injection, reaching its maximum intensity within approximately 5 s. Imaging from the ventral perspective further confirmed intense fluorescence signals accumulation in the bladder region. At 1.5 h post-injection, ex vivo imaging of harvested major organs demonstrated that fluorescence signals were predominantly localized in the kidneys (Fig. 5b). In contrast, only minimal signals were detected in the heart, liver, spleen, and lungs. These findings collectively suggest that NY-07 undergoes rapid metabolism and is primarily eliminated from the body via renal excretion. The excretion rate of NY-07 was determined by measuring its cumulative fluorescence intensity in mouse urine at various time points post-injection. The results showed that approximately 56.96 ± 3.84% of the injected dose was excreted via urine within 24 h (Fig. S8).

For comparison, we also monitored the clinically approved probe ICG, which is predominantly metabolized through the hepatobiliary system. As shown in Fig. 5c, following ICG injection, fluorescence signals predominantly accumulated in the liver region, with no significant signals observed in the kidneys or bladder. Ex vivo biodistribution imaging supported the liver-dominated metabolic pathway (Fig. 5d). Quantitative analysis the MFI in the kidney region (Fig. 5e), bladder and urine (Fig. 5f), and various organs (Fig. 5g) for NY-07 and ICG group further confirmed that NY-07, unlike ICG, is primarily clearance via the renal-bladder pathway. We evaluated the pharmacokinetic parameters of NY-07. The results (Fig. S9) showed that the clearance half-life (t_1_/_2_) of NY-07 in blood was 2.77 min, and its renal clearance rate constant was 0.25 h^−1^. The This characteristic of NY-07 can provide a suitable alternative for patients with liver dysfunction or liver tumors who are not suitable for ICG probes.

### 2.5 In vivo tumor targeting of NY-07

**Fig. 6:**
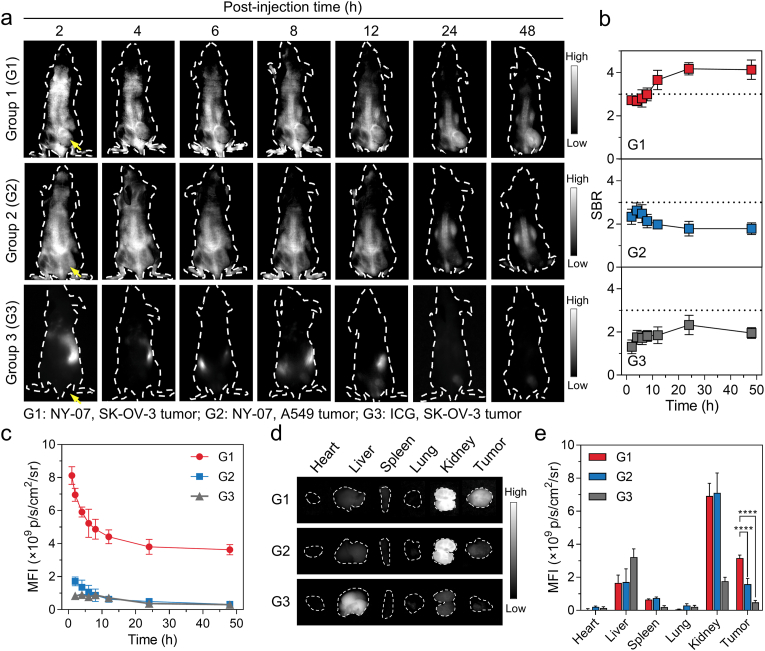
**In vivo tumor-targeted NIR-II FLI imaging of NY-07.** (a) Representative whole-body NIR-II FLI images of tumor-bearing mice, acquired over 48 h following intravenous administration of the probes. Group 1 (G1): SK-OV-3 tumor–bearing mice injected with NY-07 (10 nmol); Group 2 (G2): A549 tumor–bearing mice injected with NY-07 (10 nmol); Group 3 (G3): SK-OV-3 tumor–bearing mice injected with ICG (10 nmol). Arrows indicate tumor locations. (b) Quantification of the SBR of the tumor region relative to the contralateral thigh for each group. (c) Time-dependent changes in MFI within the tumor region for each group. (d) Representative ex vivo NIR-II FLI images of major organs and tumors harvested from mice in each group. (e) Quantification of MFI in excised organs and tumors, as determined using the imaging system's analysis software. For all imaging experiments, excitation was performed at 808 nm, and emission was collected through a 1075 nm long-pass filter. Data are presented as mean ± s.d. (n = 3).

To assess the tumor-targeting efficacy of NY-07, we established three experimental groups: SKOV-3 tumor-bearing mice administered with ICG, SKOV-3 tumor-bearing mice administered with NY-07, and A549 tumor-bearing mice administered with NY-07. Subsequently, we monitored the in vivo distribution of the NY-07 probes using an NIR-II imaging system. As illustrated in Fig. 6a, a clear fluorescence signal was detectable in the tumor region at 2 h post-injection, indicating the rapid accumulation of NY-07 in the tumor tissue. Over time, the SBR continued to increase (Fig. 6b), reaching above 3 at 6 h post-injection, which meets the clinical threshold for tumor discrimination. After 24 h, the SBR reached a plateau phase with a maximum value of 4.44. In contrast, the SBR values in tumors of A549 tumor-bearing mice injected with NY-07 and SK-OV-3 tumor-bearing mice injected with ICG remained lower than those in SK-OV-3 mice treated with NY-07 and did not exceed 3 during the 48-h observation period [2,28]. A similar trend was observed in the time-dependent changes of MFI in tumor region (Fig. 6c), ex vivo fluorescence imaging (Fig. 6d) and quantitative analysis of major organs and tumors (Fig. 6e). The MFI in tumors of SK-OV-3 mice treated with NY-07 was significantly higher than that in the A549 group or the ICG group; the ex vivo tumor fluorescence intensity in the NY-07-treated SK-OV-3 group was 1.99-fold and 6.53-fold higher than that in the A549 negative-control group and the ICG group, respectively. Additionally, we evaluated the effect of FA competitive blockade on the tumor targeting of NY-07. As shown in Fig. S10, after FA blockade, the SBR (Fig. S10b), MFI in tumor region (Fig. S10c), and ex vivo MFI (Fig. S10d and e) of tumor and main organs were significantly reduced, confirming that the tumor accumulation of NY-07 is mediated by FRα-specific targeting.

Meanwhile, biosafety assessment (Fig. S11) in vivo showed that there was no significant change in body weight of the treated mice, and H&E staining of major organs revealed no obvious pathological damage. Moreover, liver and kidney function biomarkers (Alanine aminotransferase (ALT), Aspartate aminotransferase (AST), Blood urea nitrogen (BUN), Creatinine (CREA)) were all within the normal range, collectively indicating that NY-07 possesses favorable in vivo biosafety.

### 2.6 In vivo NIR-II FLT imaging

**Fig. 7:**
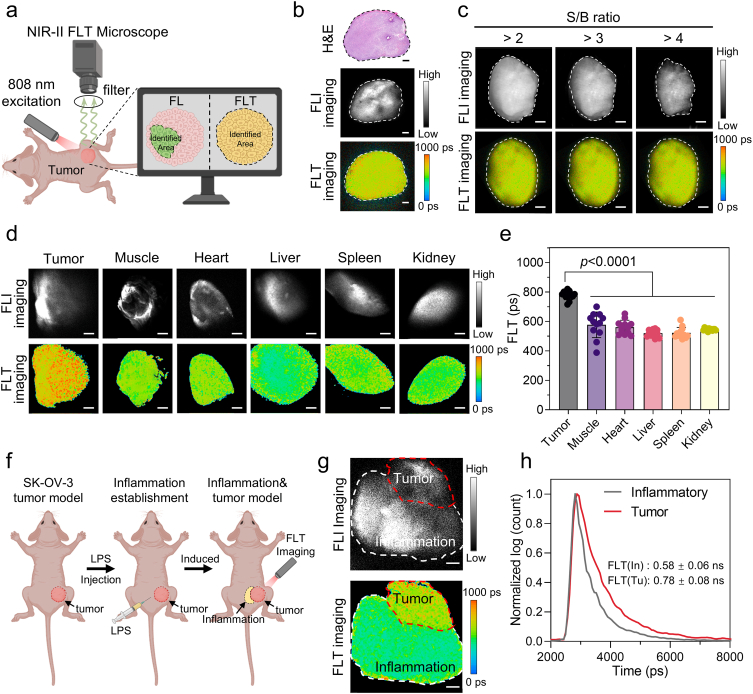
**Quantitative delineation of tumor boundaries by NIR-II FLT imaging.** (a) Schematic illustration of the tumor margin visualization using a custom-built mesoscopic NIR-II FLT microscope. (b) Representative H&E-stained histological section, NIR-II FLI, and NIR-II FLT images of tumor tissue acquired 24 h after intravenous administration of NY-07. The dotted lines indicate the tumor margins. Scale bar, 500 μm. (c) Representative FLI and FLT images of tumors displayed under different SBR thresholds, which highlight the robustness of FLT-based boundary delineation. Scale bar, 500 μm. (d) Representative NIR-II FLI and FLT images of tumor tissue, muscle tissue, heart tissue, liver tissue, spleen tissue, and kidney tissue after incubation with NY-07 (10 μM, 30 min). Scale bar, 1 mm. (e) Quantification of mean fluorescence lifetimes of tissues shown in (d). Data are presented as mean ± s.d.; statistical significance was assessed by one-way ANOVA (n = 12). (f) Schematic illustration of the tumor-adjacent chronic inflammation model and corresponding FLT imaging workflow. (g) Representative NIR-II FLI and FLT images of tumor and surrounding inflamed tissues following incubation with NY-07 (10 nM, 10 min). Scale bar, 500 μm. (h) Representative NIR-II fluorescence decay curves acquired from tumor and inflammatory regions. For all imaging experiments, λex = 808 nm and λem > 1000 nm. Data are presented as mean ± s.d.

The imaging capabilities of the NY-07 probe for NIR-II FLT in vivo were assessed in SK-OV-3 tumor-bearing mice using a custom-developed mesoscopic NIR-II FLT microscope (FLTM) (Figs. S12 and 7a). With due consideration for the operational feasibility in a clinical surgical setting, imaging was performed at 24 h post-injection. As shown in Fig. 7b, the tumor was clearly demarcated by NY-07 in the fluorescence intensity image within a 5.5 mm × 5.5 mm field of view. However, the FLI imaging showed an uneven signal distribution, which hindered accurate boundary delineation. In contrast, FLT imaging provided a uniform signal and clearly outlined the tumor contour. Quantitative analysis (Fig. S13) revealed that the tumor area measured by FLT imaging was 18.98 ± 0.17 mm^2^, significantly larger than the 12.71 ± 0.87 mm^2^ obtained by FLI imaging. Using hematoxylin and eosin (H&E) staining as the gold standard, the accuracy of tumor margin delineation achieved by FLT imaging was 91.35%, markedly higher than the 61.34% for FLI imaging, demonstrating the superior accuracy of NY-07-mediated FLT imaging in tumor margin assessment. We further examined the impact of imaging parameters on tumor boundary delineation. As shown in Fig. 7c, the tumor boundary visualized by FLI imaging varied with changes in the SBR, increasing the complexity and inconsistency of intraoperative margin determination. In contrast, FLT imaging stably and accurately displayed the tumor boundary independently of imaging parameters (Fig. S14), thereby enhancing the efficiency of intraoperative margin determination.

To further validate the specificity of NY-07-based FLT imaging, we measured the FLI and FLT signals of tumor tissue and various normal tissues (heart, muscle, liver, spleen, kidney) after incubation with the NY-07 probe. Although nonspecific adsorption resulted in detectable fluorescence signals in normal tissues (Fig. 7d), the fluorescence lifetime (Fig. 7e and S15) of tumor tissue (0.778 ± 0.029 ns) was significantly higher than that of muscle (0.577 ns), heart (0.561 ns), liver (0.520 ns), spleen (0.522 ns), and kidney (0.545 ns), showing obvious contrast in FLT imaging. The prolonged fluorescence lifetime of NY-07 in tumor tissues maybe attribute to two reasons: 1) NY-07 can specifically binds to the highly expressed FRα on the tumor cell membrane. This binding process restricts the internal rotation of the ICG derivative molecules, reduces non-radiative transitions, and thus prolongs their fluorescence lifetime [29,30]; 2) The microenvironment within tumor (such as higher viscosity and specific pH range) may also affect the fluorescence lifetime [31]. A similar result was observed in clinical cancer samples, where tumor tissues exhibited longer fluorescence lifetimes than normal tissues (Fig. S16). These results suggest that NY-07-based FLT imaging can effectively mitigate interference from nonspecific adsorption in tumor recognition.

Subsequently, we established a tumor-adjacent chronic inflammation model in mice via repeated local injections of lipopolysaccharide (LPS) (Fig. 7f) to evaluate the ability of NY-07 to distinguish tumor from inflammatory tissue. After incubation with 10 nM NY-07 for 10 min, FLT microscopy showed that both tumor and inflamed regions exhibited FLI signals, making them indistinguishable by FLI imaging (Fig. 7g). However, FLT imaging revealed a discernible boundary between the tumor and inflammatory regions: the tumor area appeared yellow, whereas the inflamed area appeared green, further highlighting the advantage of NY-07-mediated FLT imaging in tumor boundary delineation. Fluorescence decay analysis (Fig. 7h) indicated that the mean fluorescence lifetime in the tumor region (0.78 ± 0.08 ns) was longer than that in the inflammatory region (0.58 ± 0.06 ns). This difference may be attributed to the high expression of FRα in ovarian cancer, which specificity bonded to the probe and prolongs its fluorescence lifetime, whereas inflammation-associated regions showed low expression of FRα (mainly expressing FRβ) [27]., where the probe is primarily nonspecifically adsorbed, resulting in a smaller lifetime shift. The developed NIR-II fluorescence lifetime-targeted small-molecule probe NY-07 effectively distinguishes tumor from inflammatory tissue, which can help reduce false-positive signals caused by inflammation during surgery and improve the accuracy of tumor margin determination.

## 3 Conclusion

In conclusion, this study establishes a clinically translatable strategy for precise tumor margin delineation by integrating renal-clearable probe engineering, active targeting, and NIR-II FLT imaging. This approach overcomes the inherent limitations of conventional intensity-based guidance in complex surgical environments. Exploiting lifetime-encoded contrast that is intrinsically independent of probe concentration and tissue optical heterogeneity, this platform enables accurate discrimination among malignant, inflammatory, and normal tissues, allowing reliable intraoperative navigation and complete tumor resection. The platform demonstrates excellent pharmacokinetic properties, high biosafety, and real-time imaging capabilities, underscoring its significant translational potential. Together, these findings validate NIR-II FLT imaging as a robust tool for precision surgical oncology and offer a versatile framework for advancing next-generation molecular probes and image-guided therapeutic strategies.

## 4 Materials and methods

### 4.1 Materials

Fetal bovine serum (FBS), penicillin-streptomycin solution, trypsin, and PBS were procured from Gibco. McCoy's 5A medium was provided by VivaCell. ICG was obtained from Nanjing Haina Pharmaceutical Co., Ltd., and FA was acquired from Sigma-Aldrich (USA). All other analytical-grade reagents were supplied by Aladdin and utilized without further purification unless otherwise specified.

### 4.2 Cell culture

The SK-OV-3 and A549 cell lines were obtained from Procell (Wuhan, China). All cell-handling procedures, including thawing, subculturing, and cryopreservation, were conducted under strict aseptic conditions.

### 4.3 Synthesis and structural characterization of NY-07

NY-07 was synthesized according to previously reported literature [27]. All synthetic procedures were conducted under an inert atmosphere, with key intermediates and the product purified via HPLC. The validity of NY-07 was verified by MALDI-TOF (SYNAPT G2-Si) and ^1^H NMR spectroscopy (AVANCE III 400 MHz, Bruker).

### 4.4 UV-vis-NIR absorption spectroscopy

NY-07 was prepared as a solution in 0.9% NaCl (10 μg/mL). The UV-Vis-NIR absorption spectroscopy were recorded in spectrophotometer (UV-2600, Shimadzu) across the wavelength range of 325–900 nm. For presentation purposes, the obtained data were normalized.

### 4.5 NIR-II fluorescence spectroscopy

The fluorescence spectra were recorded using a steady-state/transient fluorescence spectrometer (FSL920, Edinburgh Instruments, UK). The excitation was 660 nm and emission was measured over the range of 700 to 1300 nm. NY-07 was prepared in a 0.9% NaCl saline solution at a concentration of 1 μg/mL.

### 4.6 Measurement of fluorescence intensity of NY-07 and ICG

Equal molar concentrations of NY-07 (20 μg/mL) and ICG (12 μg/mL) solutions were prepared in PBS (pH 7.4). Subsequently, 300 μL aliquots of each sample were transferred into three separate wells of a transparent microplate (Corning) and imaged using a in vivo imaging system (AniView 100 Phoenix, BLT). Excitation was carried out at 808 nm, and fluorescence emission was captured in the NIR-I area (820 ± 20 nm) and NIR-II area (>1075 nm, long-pass filter) channels under identical exposure settings. All measurements were performed in triplicate. Fluorescence intensity was quantified with the integrated software (BLT Analysis), and results are expressed as mean ± standard deviation.

### 4.7 NIR-II FLT imaging measurement

NIR-II FLT imaging of NY-07 and ICG was conducted using a custom-built FLT imaging microscope (5.5 mm × 5.5 mm, 2.5 μm/pixel). In brief, 1 μg/mL NY-07 and ICG were prepared in ultrapure water and excited with an 808 nm fs laser (Ultra I, Coherent). The emitted fluorescence signals (λe> 1000 nm) were collected and recorded with a TCSPC module (FT1040, Simics Inc.). The fluorescence decay profiles were fitted using a single-exponential model. The fluorescence lifetime value is obtained based on the formula: f(t) = ae^(-t/τ). The raw TCSPC data were processed and analyzed in MATLAB R2025a to extract the NIR-II fluorescence lifetimes for both NY-07 and ICG.

### 4.8 In vitro photobleaching stability assessment

NY-07 and ICG were prepared as aqueous solutions in ultrapure water at a concentration of 1 μg/mL. Then, 200 μL solution was dispensed into individual wells of a clear-bottom microplate (Corning). The samples were subjected to continuous illumination using an 808 nm laser (FC 808 10W) operating at a fixed power density of 400 mW/cm^2^. Irradiation was performed for designated durations: 0, 30, 60, 90, 120, and 150 s. Immediately following each irradiation period, fluorescence imaging was conducted. Excitation wavelength was 745 nm and emission at 820 nm, with the exposure time maintained consistently throughout the experiment. MFI were quantified with the system software (Living Image). The photobleaching rate was calculated relative to the intensity of the initial sample (t = 0 s). The photobleaching decay curve was plotted using GraphPad Prism software, and the half-life (t_1_/_2_) was calculated by nonlinear fitting to quantify photostability.

### 4.9 Storage stability test

NY-07 and ICG were dissolved in ultrapure water to 10 μg/mL and stored at 4 °C in the dark. Collect samples at 0, 1, 3, 5, and 7 days and measure their fluorescence intensity using a fluorescence imaging system (IVIS Lumina, PerkinElmer) under the same imaging parameters. Storage stability was calculated relative to the fluorescence intensity on day 0 (set as 100%), and time-dependent relative fluorescence changes curves were plotted using Origin software.

### 4.10 Stability in different solvents

NY-07 and ICG were separately dissolved in three clinically relevant media: 5% glucose injection, 0.9% NaCl saline, and pH 7.4 PBS with a concentration of 200 μg/mL. The solutions kept at room temperature protected from light. Optical imaging of virous samples were recorded at 0, 1, 2, and 3 days after preparation.

### 4.11 Penetration performance test

NY-07 and ICG were prepared as 10 nmol/mL solutions (13.1 μg/mL for NY-07, 7.75 μg/mL for ICG) and loaded into glass capillaries (inner diameter 1 mm) placed at the bottom of 35 mm culture dishes. Different thicknesses of 1% intralipid phantom were layered over the capillaries. The NIR-I and NIR-II fluorescence imaging was conducted using a full-spectrum in vivo imaging system (AniView 100 Phoenix, BLT). For NIR-I imaging, excitation was 745 nm and emission was 820 nm. NIR-II imaging utilized 808 nm excitation and collection through 1075 nm LP filter. All experiments were replicated 3 times.

### 4.12 Confocal microscopy imaging

SKOV-3 and A549 cells (1.5 × 10^4^ cells per well) were seeded in confocal dishes and incubated for 24 h. Following removal of the original culture medium, cells were subjected to three distinct treatment conditions: an experimental group (serum-free medium supplemented with 30 nM NY-07), a competitive-block group (serum-free medium containing 30 nM NY-07 plus 3 mM free FA), and a negative-control group (A549 cells cultured in serum-free medium with 30 nM NY-07). After a 2 h incubation period, cells were washed three times with PBS and imaged through a confocal microscope (STELLARIS 5, Leica). Excitation was 638 nm and emission was 750–850 nm.

### 4.13 Flow cytometry analysis

SKOV-3 and A549 cells (1 × 10^5^ cells) were seeded in 6-well plates a and incubated for 24 h. Following medium removal and a single wash with PBS, serum-free medium with 30 nM NY-07 was added. In the FA blocking group, an additional 3 mM free FA was introduced. After a 2-h incubation, cells were washed, detached and harvested for flow cytometry analysis. Samples were analyzed on a CytoFLEX LX flow cytometer (excitation: 800 nm, emission: 820 ± 20 nm). Data were quantified using FlowJo v10.8.1 software (n = 3).

### 4.14 Cell toxicity assessment

The in vitro cytotoxicity of the NY-07 probe was evaluated using the standard CCK-8 assay. SK-OV-3 (FRα-high) and A549 (FRα-low) cells at the logarithmic growth phase were seeded into 96-well plates at a density of 5 × 10^3^ cells per well and cultured for 24 h to allow cell attachment. After removing the supernatant, fresh medium containing various concentrations of NY-07 (0, 3, 6, 12, 25, 50, 100, and 200 μM) was added to the wells, with four replicate wells per concentration. Control wells (medium only, no probe) and blank wells (medium only, no cells) were also included. Following a 24-h incubation, the medium was replaced 100 μL fresh medium and 10 μL of CCK-8 solution was added to each well. After incubated for an additional 2 h in the dark. The absorbance at 450 nm was measured using a microplate reader, and cell viability was calculated. The experiment was repeated three times, and data are presented as mean ± standard deviation to evaluate the biocompatibility and safe concentration range of NY-07.

### 4.15 In vivo biodistribution imaging

Healthy BALB/c mice were shaved on the dorsal and ventral sides 24 h before experiments. Mice received tail-vein injections of equimolar doses (10 nmol) of NY-07 (131 μg/mL, 100 μL) or ICG (77.1 μg/mL, 100 μL). Immediately after injection, mice were placed in an NIR-II in vivo imaging system, and fluorescence images of dorsal or ventral position were acquired at preset time points. Excitation was 808 nm, and emission were collected through a long-pass filter with a cutoff above 1000 nm, with an exposure duration set to 0.5 s. At 1.5 h following injection, the mice were euthanized and main organs (heart, liver, spleen, lungs, and kidneys) were harvested. These organs were briefly washed with ice-cold PBS, positioned in culture dishes, and subsequently imaged under identical conditions. The remaining mice were placed in metabolic cages, and urine were obtained at 24 h post-injection. These urine samples were subsequently transferred into 1 mL EP tubes for NIR-II imaging. To determine the mass and metabolic efficiency of NY-07 at various time points, a standard curve of fluorescence intensity versus NY-07 concentration was generated using a dilution series of the probe in PBS. The mass of NY-07 metabolized was subsequently obtained from this standard curve.

### 4.16 In vivo tumor-targeted evaluation

Mouse models bearing tumors with high FRα expression (SKOV-3) and low FRα expression (A549) were established. Four experimental groups were designated: 1) SKOV-3 tumor-bearing mice administered with ICG (10 nmol); 2) SKOV-3 tumor-bearing mice administered with NY-07 (10 nmol); 3) SKOV-3 tumor-bearing mice co-administered with NY-07 (10 nmol) and FA (2 mg); 4) A549 tumor-bearing mice administered with NY-07 (10 nmol). When tumor volume reached approximately 200 mm^3^, the mice was injected with different probe solutions. Then, the NIR-II fluorescence imaging was recorded at 2, 4, 6, 8, 12, 24, and 48 h post-injection. Imaging parameters included an 808 nm excitation wavelength, a long-pass emission filter (>1075 nm), and a fixed exposure time of 0.25 s. Following the final imaging time point, mice were euthanized. Heart, liver, spleen, lungs, kidneys and tumors were harvested, and subjected to NIR-II imaging for biodistribution analysis. The MFI within tumor regions and organs was quantified using the system's proprietary software.

After the imaging process was completed, the blood of mice was collected for biochemical analysis and the main organs was exfoliated for H&E staining to evaluate the in vivo biocompatibility of NY-07.

### 4.17 In vivo NIR-II FLT imaging

Nude mice bearing SK-OV-3 tumors with volumes of approximately 100 mm^3^ were administered 10 nmol NY-07. At 24 h post-injection, mice were anesthetized using isoflurane. The tumor region was then subjected to simultaneous FLI and FLT imaging via FLIM. Subsequently, the tumor and major organs were surgically excised for NIR-II FLI and FLT imaging. Following imaging, tumor tissues were fixed for H&E staining. FLI imaging was quantified using the instrument's software. FLT imaging data were processed, quantified, and analyzed using MATLAB 2025a.

### 4.18 Establishment of chronic inflammation model and NIR-II FLT imaging

In this study, SK-OV-3 tumor-bearing mice were utilized. Following anesthesia, lipopolysaccharide (LPS) was administered subcutaneously around the tumor at a dose of 3 μg per gram of body weight, with injections repeated every two days for a total of three doses. The chronic inflammation model was deemed established when the injection sites exhibited no significant signs of redness, swelling, suppuration, or exudation. Subsequently, mouse received 10 nmol NY-07. Imaging was conducted 24 h post-injection using NIR-II FLT imaging system. The fluorescence lifetime within the tumor and inflammation regions were quantified using the corresponding software.

### 4.19 FLT imaging of clinical samples

Clinical samples collection was conducted in strict compliance with the ethical tenets of the Declaration of Helsinki and received formal approval from the medical ethics committee of nanjing drum tower hospital (No. 2024-673-01). Written informed consent, which detailed the use of tissue specimens and clinical information for scientific research, was secured from all patients before surgery. Furthermore, all patient data were thoroughly anonymized prior to any data compilation and analytical processes to safeguard privacy.

Lung adenocarcinoma (LADC) specimens were surgically excised from ten patients. Following resection, the tissue samples were incubated with NY-07 (10 nM) at ambient temperature for a duration of 10 min. After incubation, the samples were washed with PBS 3 times to remove unbound reagent. Finally, the prepared clinical specimens were subjected to imaging using a customized NIR-II FLT mesoscopic system.

### 4.20 Statistical analysis

Dates are expressed as mean ± s.d. For statistical test, a minimum sample size of three (n ≥ 3) was used. Two-tailed student's t-test was used to analyse comparisons of continuous data between different treatment groups. Statistical significance was defined as p < 0.05, with specific thresholds indicated as ∗p < 0.05, ∗∗p < 0.01, and ∗∗∗p < 0.001.

## CRediT authorship contribution statement

**Jin Zhang:** Data curation, Formal analysis, Funding acquisition, Writing – original draft. **Jiuling Liao:** Data curation, Formal analysis. **Dong Han:** Resources. **Xingsheng Ren:** Data curation, Resources. **Dehong Hu:** Funding acquisition, Validation. **Duyang Gao:** Formal analysis, Resources. **Pengfei Zhang:** Formal analysis, Visualization. **Shengnan Yuan:** Supervision. **Wei Zheng:** Formal analysis, Resources. **Christopher J. Butch:** Resources. **Bo Dai:** Resources. **Huiming Cai:** Resources. **Yiqing Wang:** Conceptualization, Resources. **Hairong Zheng:** Funding acquisition. **Zonghai Sheng:** Conceptualization, Funding acquisition, Writing – review & editing.

## Declaration of competing interest

The authors declare that they have no known competing financial interests or personal relationships that could have appeared to influence the work reported in this paper.

## Data Availability

Data will be made available on request.
