# Supplementary material for: A renally clearable tumor-targeted probe enabling functional delineation of tumor margins by NIR-II fluorescence lifetime imaging
Source: Mater Today Bio. 2026 May 23;38:103274. doi: 10.1016/j.mtbio.2026.103274 (PMC13227218; doi:10.1016/j.mtbio.2026.103274)
Supplement: Multimedia component 1 [file mmc1.docx]

Supporting Information

A Renally Clearable Tumor-Targeted Probe Enabling Functional Delineation of Tumor Margins by NIR-II Fluorescence Lifetime Imaging

Jin Zhang^1,2†^, Jiuling Liao^2,3†^, Dong Han^4†^, Xingsheng Ren^2,3^, Dehong Hu^1,2^, Duyang Gao^1,2^, Pengfei Zhang^5^, Shengnan Yuan^1,2^, Wei Zheng^2,3^, Christopher J. Butch^4^, Bo Dai^7^, Huiming Cai^4,6*^, Yiqing Wang^4*^, Hairong Zheng^2^, Zonghai Sheng^1,2*^

^1^Research Center for Advanced Detection Materials and Medical Imaging Devices, Institute of Biomedical and Health Engineering, Shenzhen Institute of Advanced Technology, Chinese Academy of Sciences, Shenzhen 518055, P. R. China.

^2^State Key Laboratory of Biomedical lmaging Science and System, Shenzhen 518055, P. R. China.

^3^Research Center for Biomedical Optics and Molecular Imaging, Shenzhen Key Laboratory for Molecular Imaging, Guangdong Provincial Key Laboratory of Biomedical Optical Imaging Technology, Shenzhen Institutes of Advanced Technology, Chinese Academy of Sciences, Shenzhen 518055, P. R. China

^4^Department of Biomedical Engineering, College of Engineering and Applied Sciences, State Key Laboratory of Analytical Chemistry for Life Science, Nanjing University, Nanjing, P. R. China

^5^Guangdong Key Laboratory of Nanomedicine, Chinese Academy of Sciences-Hong Kong Joint Lab for Biomaterials, Chinese Academy of Sciences Key Laboratory of Biomedical Imaging Science and System, Institute of Biomedicine and Biotechnology, Shenzhen Institutes of Advanced Technology, Chinese Academy of Sciences, Shenzhen, 518055, P. R. China.

^6^Nanjing Nuoyuan Medical Devices Co., Ltd, Nanjing, P. R. China

^7^Department of Thoracic Surgery, Nanjing Drum Tower Hospital, School of Medicine, Nanjing University, Nanjing, P. R. China

^†^These authors contributed equally to this work.

* Corresponding author:

E-mail address: [caihuiming@nuoyuanmedical.cn](mailto:caihuiming@nuoyuanmedical.cn) (Huiming Cai); [wangyiqing@nju.edu.cn](mailto:wangyiqing@nju.edu.cn) (Yiqing Wang); zh.sheng@siat.ac.cn (Zonghai Sheng)

**Supporting Figures**


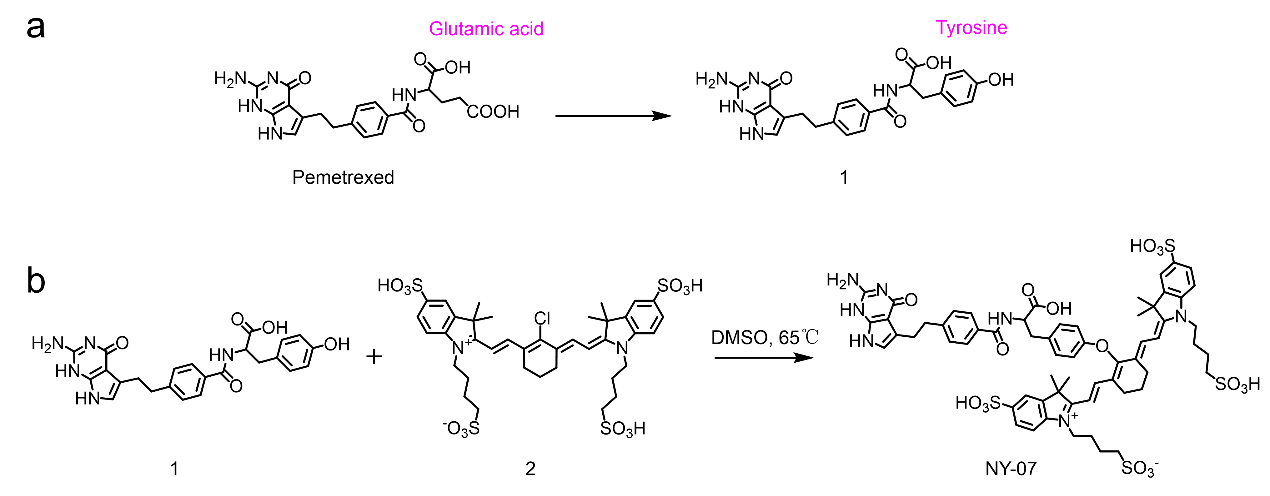


Figure S1. Schematic illustration of the preparation of NY-07. (a) Structure of the targeting molecule precursor **1**, designed by substituting glutamic acid in the FDA-approved drug pemetrexed with tyrosine. (b) Synthesis route of NY-07: compound **1** reacts with cyanine derivative **2** in DMSO at 65°C for 2 days.


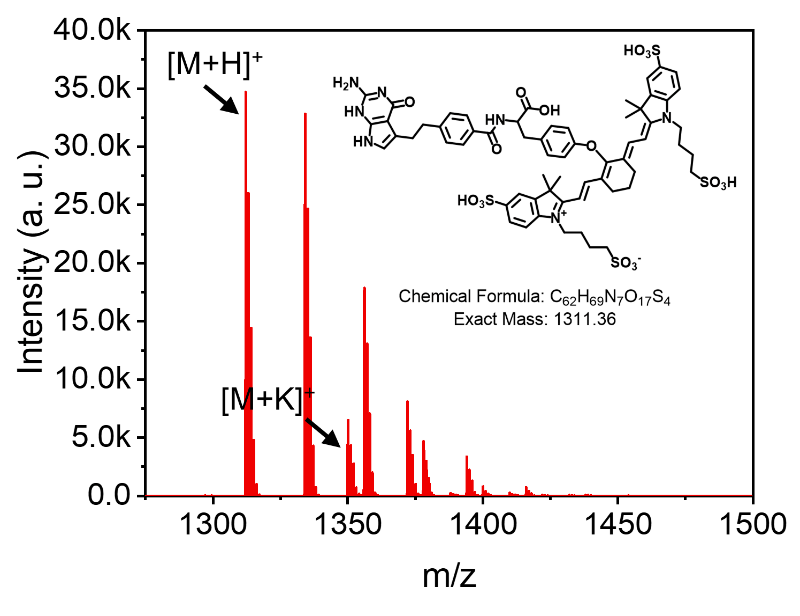


Figure S2. MADI-TOF mass spectrometry of NY-07. [M+H]^+^ =1312.116, [M+K]^+^ =1350.065. The subsequent peaks at equal mass-to-charge ratio intervals of 22 correspond to sodium ions replacing hydrogen atoms on the sulfonate groups.


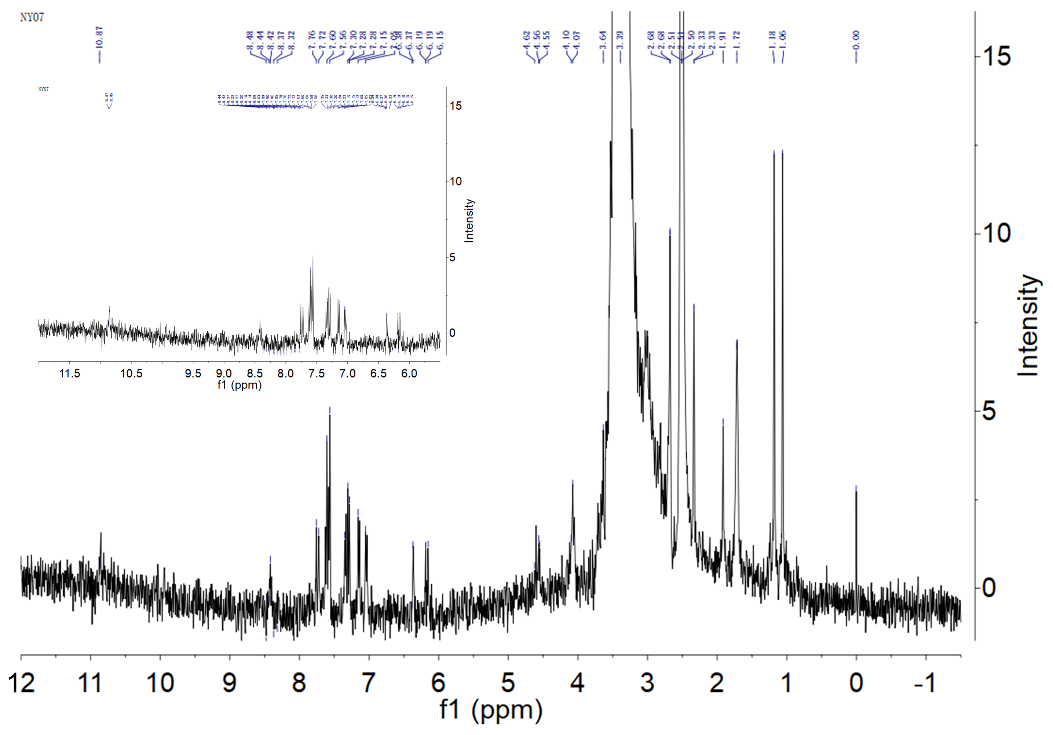


Figure S3. ^1^HNMR spectrum of NY-07 in DMSO-d6.


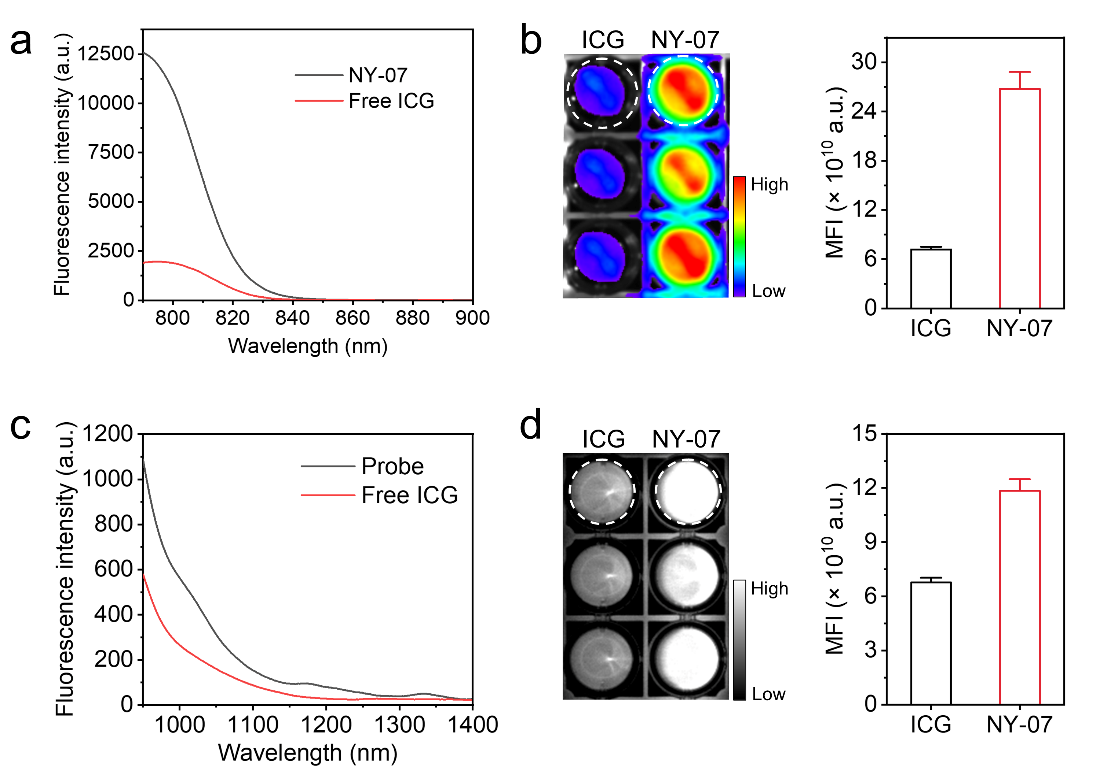


Figure S4. Fluorescence spectra and FLI imaging of NY-07 and ICG in the NIR-I and NNIR-II windows. (a, b) Fluorescence spectrum, imaging, and quantitative region-of-interest (ROI) analysis of NY-07 and ICG in the NIR-I window. (c, d) Fluorescence spectrum, imaging, and quantitative ROI analysis of NY-07 and ICG in the NIR-II window. The concentrations of NY-07 and ICG are 15 µM.


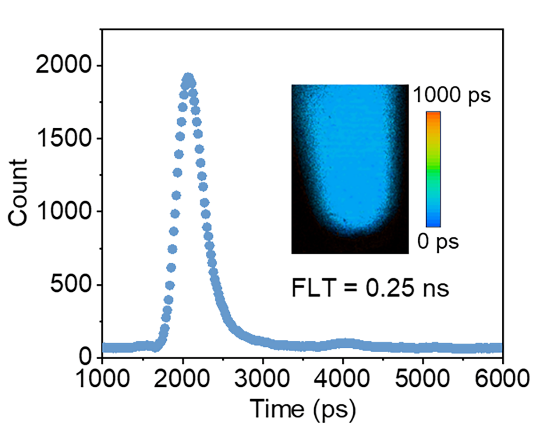


Figure S5. NIR‑II fluorescence decay curve of a 10 μg/mL ICG solution. Inset shows the corresponding two‑dimensional FLT image (Excitation: 808 nm; Detection: >1000 nm).


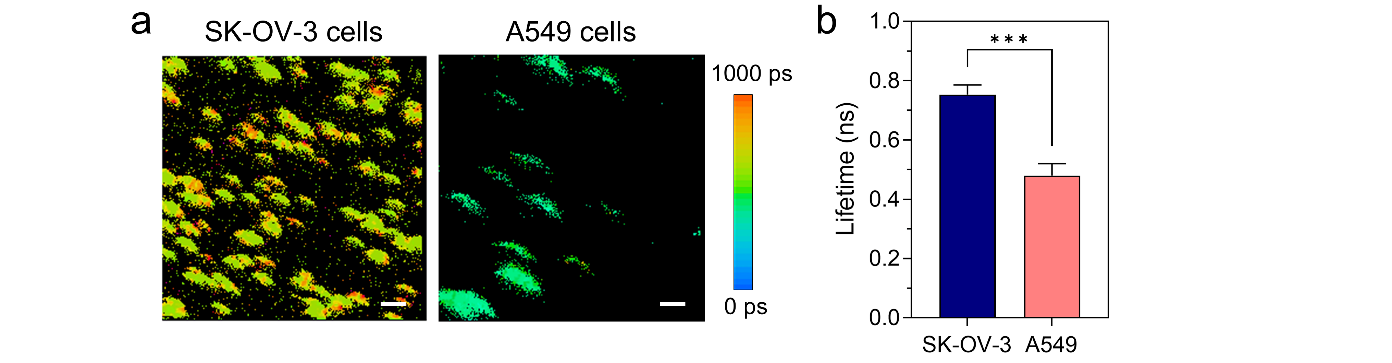


Figure S6. (a) Fluorescence lifetime imaging of SK-OV-3 and A549 cells after incubation with NY-07 for 12 h. Scale bar: 20 μm. (b) Fluorescence lifetimes of cell suspensions of SK-OV-3 and A549 cells after incubation with NY-07 for 12 h. Probe concentration: 20 μM.


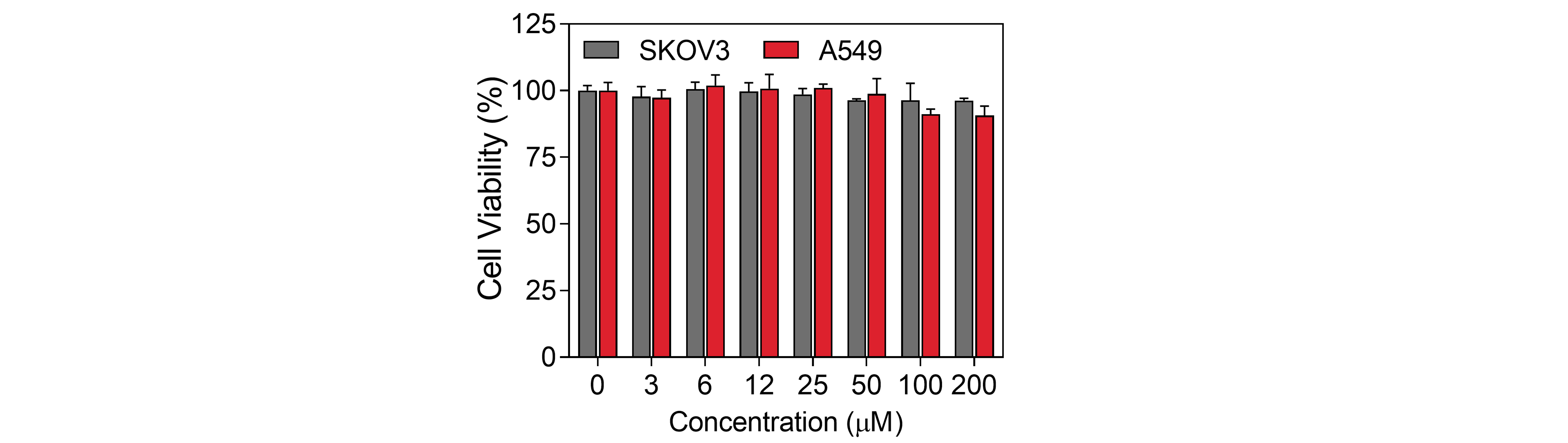


Figure S7. Cell viability of SK-OV-3 and A549 after treatment with different concentrations of NY-07.


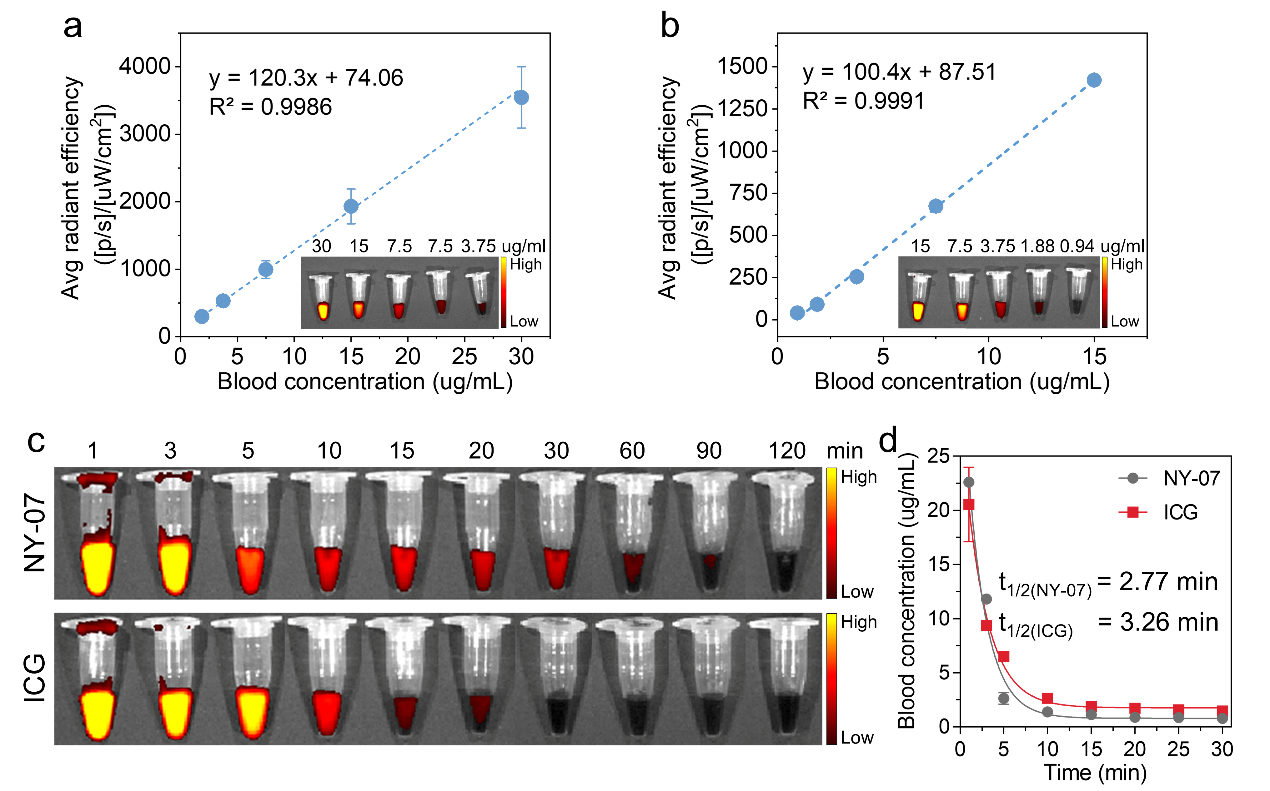


Figure S8. In vivo pharmacokinetic study of NY-07. (a, b) Standard curves showing the relationship between different concentrations of NY-07 and ICG in blood and their corresponding fluorescence intensities. Insets show fluorescence images (Excitation: 745 nm; Emission: 820 nm). (c) Fluorescence intensity images of mouse blood samples at different time points. A 5 µL aliquot of blood containing anticoagulant was dissolved in 45 µL of PBS. (d) Pharmacokinetic curves of NY-07 and ICG.


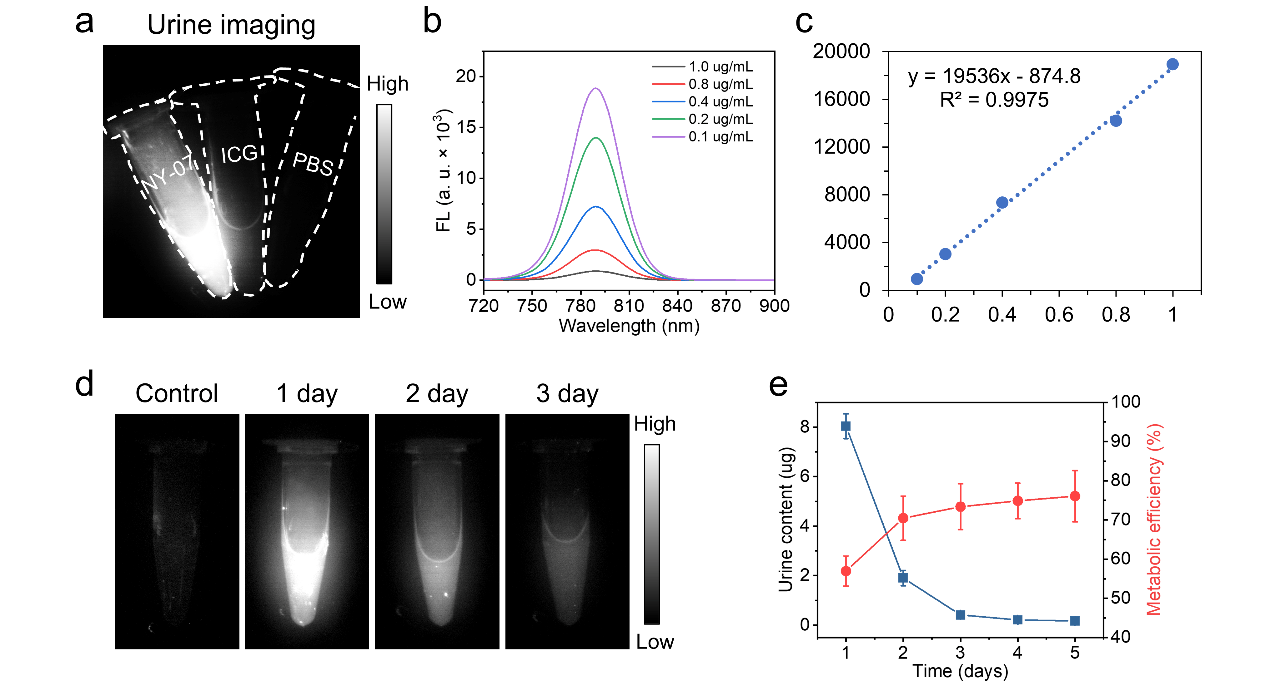


Figure S9. (a) Fluorescence intensity images of mouse urine collected at 24 h post-injection from different treatment groups. (b and c) Fluorescence spectra of NY-07 in PBS solution at various concentrations and the corresponding function of concentration vs fluorescence intensity. (d) Fluorescence intensity images of mouse urine collected at different time points after injection of 10 nmol NY-07. (e) The amount and corresponding excretion rate of NY-07 metabolized in urine at different time points.


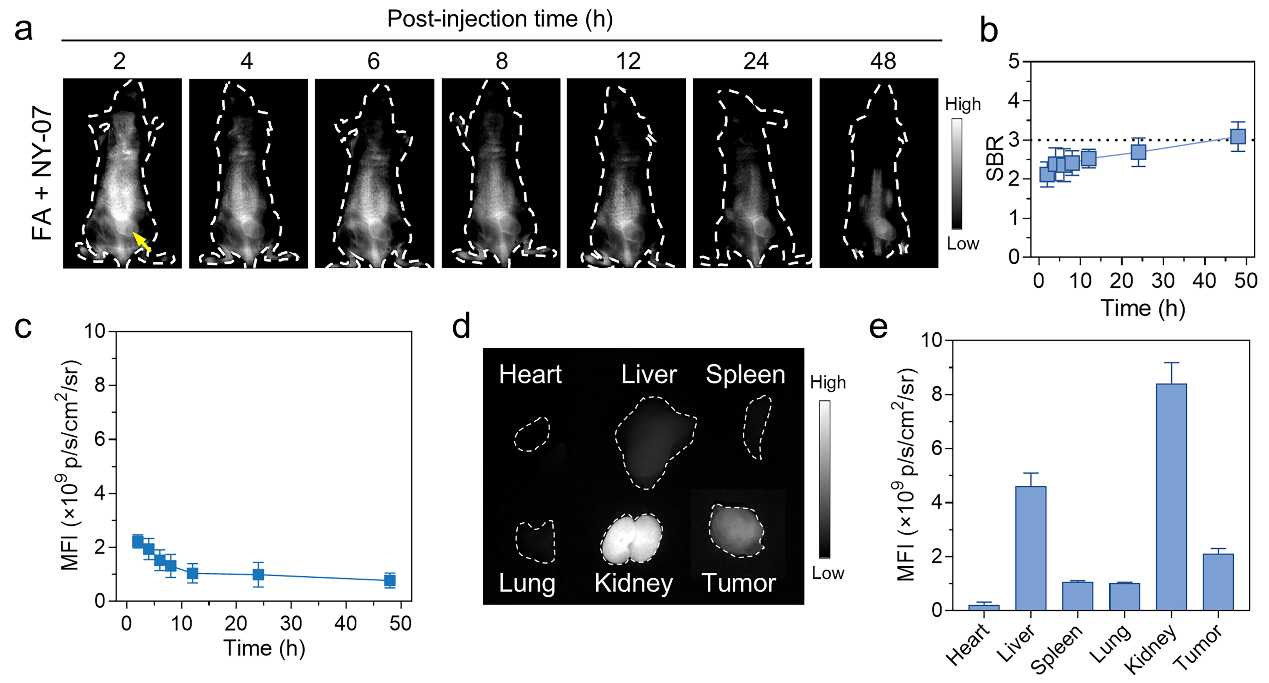


Figure S10. (a) NIR‑II fluorescence imaging of the FAX blocking group within 48 h after NY‑07 injection. Arrows indicate the tumor region. (b) SBR of the tumor area over time. (c) Time‑dependent changes in the MFI in the tumor region. (d) Ex vivo fluorescence imaging of major organs and tumors. (e) Quantitative analysis of the MFI of major organs and tumors from (d).

Figure S11. In vivo biosafety assessment. (a) H&E-stained sections of major organs (heart, liver, spleen, lung, and kidney) from mice at 48 h after injection of PBS or NY-07. Scale bar is 100 µm. (b) Biochemical analysis of serum liver and kidney function parameters (ALT, AST, BUN, CREA).


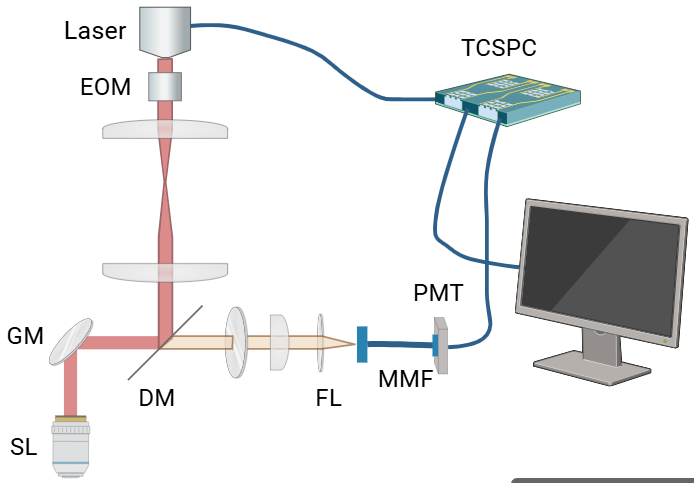


Figure S12. Schematic diagram of the custom-built microscope system for NIR-II FLI and FLT imaging

Figure S13. Detectable tumor area in H&E-staining, NIR-II FLI, and NIR-II FLT images from Figure 7b.

Figure S14. The mean signal area of FLI imaging and FLT imaging under different SBR conditions.


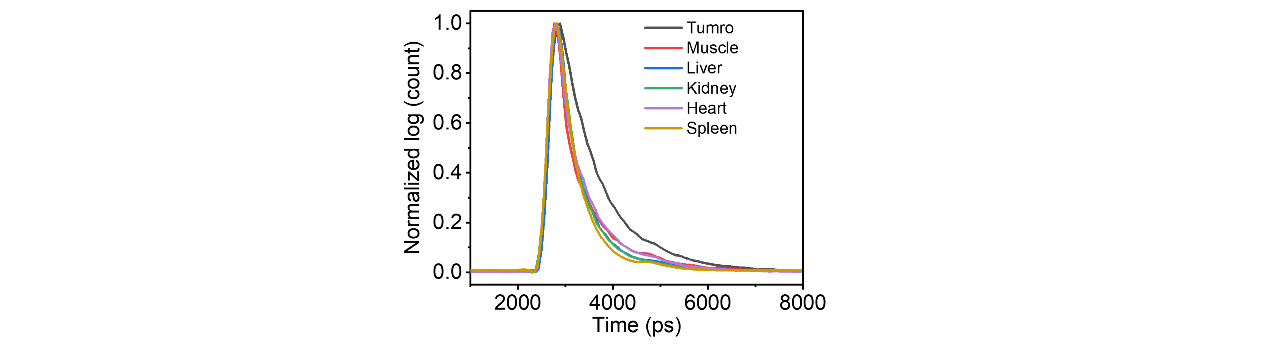


Figure S15. The NIR-II fluorescence decay curves of various organs and tumors after incubation with NY-07 under continuous 808 nm laser irradiation. The concentration of NY-07 was 10 nM.


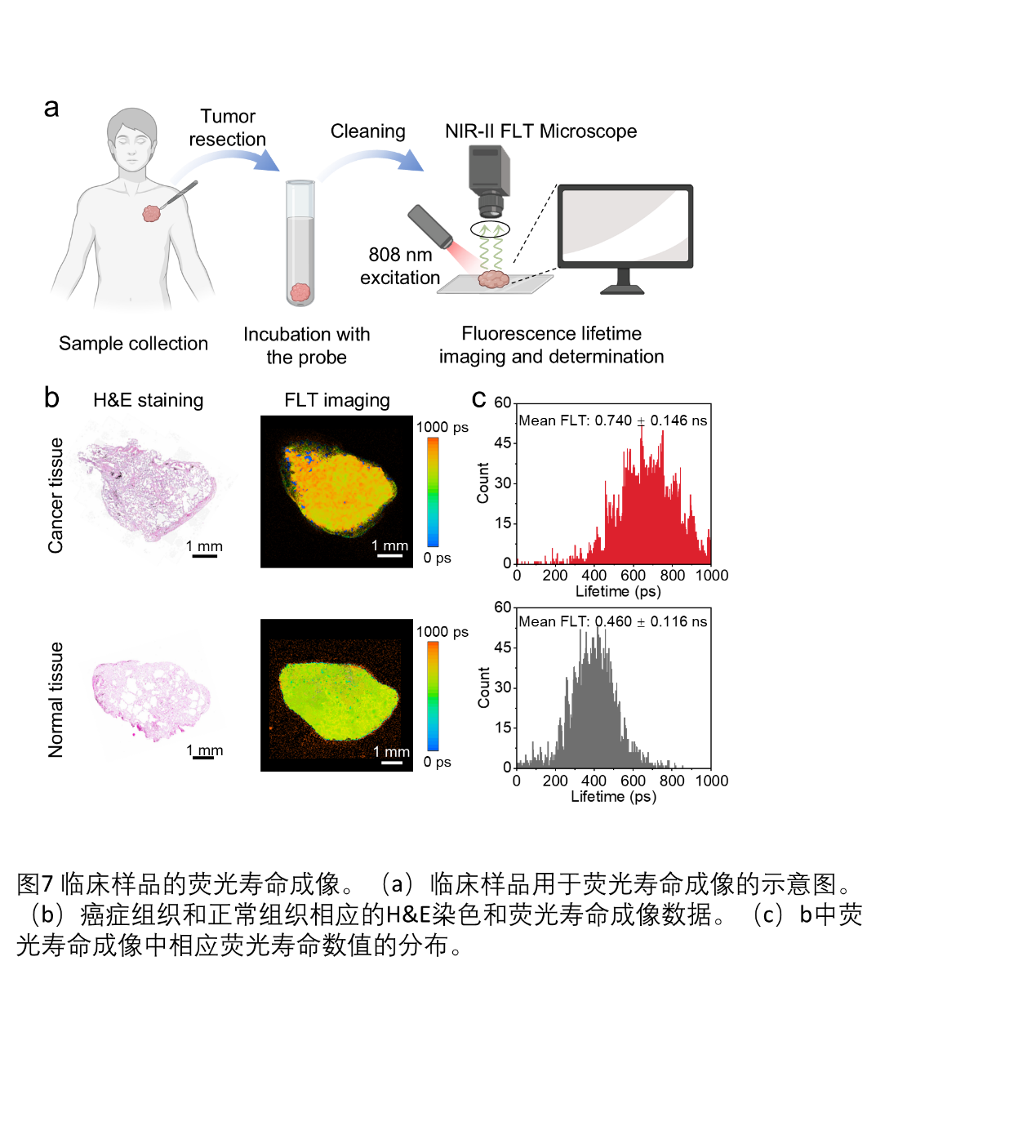


Figure S16. Fluorescence lifetime imaging of clinical samples. (a) Schematic illustration of FLT imaging for rapid clinical tumor identification. (b) Corresponding H&E staining and FLT imaging of cancer and normal tissues. (c) Distribution of fluorescence lifetime values derived from the FLT imaging shown in (b). λex = 808 nm and λem > 1000 nm. Probe concentration is 10 nM.
